# Human Lung Mast Cells as a Possible Reservoir for Coronavirus: A Novel Unrecognized Mechanism for SARS-CoV-2 Immune-Mediated Pathology

**DOI:** 10.3390/ijms25126511

**Published:** 2024-06-13

**Authors:** Rebecca Praetzel, Chris Kepley

**Affiliations:** Molecular and Cellular Sciences, Liberty University College of Osteopathic Medicine, Lynchburg, VA 24502, USA

**Keywords:** COVID-19, renin source, TMPRSS2, ACE2, SARS-CoV-2, human mast cells

## Abstract

The pathogenic severe acute respiratory syndrome coronavirus 2 (SARS-CoV-2) is a global health concern. Cell entry of SARS-CoV-2 depends on viral spike (S) proteins binding to cellular receptors (ACE2) and their subsequent priming by host cell proteases (TMPRSS2). Assessing effects of viral-induced host response factors and determining which cells are used by SARS-CoV-2 for entry might provide insights into viral transmission, add clarity to the virus’ pathogenesis, and possibly reveal therapeutic targets. Mast cells (MCs) are ubiquitously expressed tissue cells that act as immune sentinels given their ability to react specifically to pathogens at environmental interfaces, such as in the lung. Several lines of evidence suggest a critical role for MCs in SARS-CoV-2 infections based on patients’ mediator profiles, especially the “cytokine storm” responsible for most morbidity and mortality. In this pilot study, we demonstrated that human lung MCs (*n* = 3 donors) are a source of renin and that they upregulate the membrane receptor for SARS-CoV-2 (ACE2) as well as the protease required for cellular entry (TMPRSS2) under certain conditions. We hypothesized that infection of human MCs with SARS-CoV-2 may be a heretofore-unrecognized mechanism of viral pathogenesis, and further studies are required to assess this question.

## 1. Introduction

The SARS-CoV-2 RNA virus, first identified in 2019 in Wuhan, China, is associated with multiple respiratory diseases that can lead to profound morbidity and mortality of those infected. Presently, human COVID-19 viruses are evolving due to their high genomic nucleotide substitution rates, recombination, and urbanization. Coronavirus can cause respiratory syndrome, which as of February 2024 has infected over 700,000,000 worldwide and has caused over 7,000,000 deaths according to the World Health Association. Given the significance of this virus, the emergence of more virulent strains, and an unclear understanding of the pathological mechanisms it induces in humans, more studies are needed to understand the effects of the virus, especially in the lung, so that critical questions can be answered to help guide therapeutic and diagnostic strategies. A basic question that still needs to be answered involves what cells in the respiratory system serve as a reservoir for the virus. Also, questions still remain as to how SARS-CoV-2 infection leads to the inflammatory mediator milieu that ultimately leads to morbidity and mortality associated with the virus. Although vaccines have been available recently, the coronavirus will likely remain a significant problem as it mutates and continually evades our efforts to eradicate it.

Mast cells (MCs) are ubiquitously expressed in tissues and are uniquely able to initiate and propagate certain inflammatory responses that provide an interface between innate and adaptive immunity [1]. They are activated through many pathways (FcεRI and non-FcεRI pathways) to release pre-formed mediators (e.g., histamine, chymase, tryptase, TNF-α), newly synthesized arachidonic acid metabolites, as well as newly made cytokines (especially GM-CSF) and proteases (chymase, tryptase, and possibly renin). Mast cell granules are unique in that they contain pre-made TNF-α, which can be released immediately upon FcεRI activation [1]. Human MCs also produce other potential viral mediators, including reactive oxygen species, leukotrienes, prostaglandin D2 (PGD2), IL-9, and heparin [2]. In short, MCs occupy a critical niche at the interface of innate and acquired immunity in the lung, and may function to perturb or help to restore homeostasis, which can either promote health or contribute to disease.

There are several lines of evidence to suggest a critical role for MCs in SARS-CoV-2 infections. One study investigated changes in several cytokines in serum in COVID-19 patients [3]. Initial plasma IL-1, IL 6, IL-8, IL-9, IL-10, GM-CSF, IFN, MIP, PDGF, and TNF-α concentrations were higher in COVID-19 patients than in healthy adults. Further comparison between intensive care unit (ICU) and non-ICU patients showed that plasma concentrations of IL-2, IL-7, IL-10, MCP1, MIP, and TNF-α were higher in ICU patients than in non-ICU patients [3]. The hallmark of COVID-19 pathogenesis is the cytokine storm with elevated levels of IL-1, IL-6, TNF-α, and GM-CSF [4]; all of these cytokines are produced by human MCs, as we and others have shown [4]. Virus-activated MCs produce histamine, prostaglandin D2, IL-1, IL-6, and leukotriene C4, all of which induce bronchoconstriction and lung inflammation, a hallmark for symptoms of COVID-19 [5]. It has been proposed that the mechanism of action of famotidine (a histamine blocker) for COVID-19 patients involves on-target histamine receptor H2 activity, and that development of clinical COVID-19 involves dysfunctional MC activation and histamine release [3,6]. Interestingly, in 59 COVID-19 hospitalized patients, histological findings showed MC hyperplasia (100%), superficial perivascular infiltrate of lymphocytes (94%), and superficial edema (47%), all cutaneous features related to a MC-mediated vascular leak process [7]. Post-mortem lung biopsies of COVID-19 patients showed a significantly increased density of MCs [8]. Lastly, MCs uniquely express the serine protease tryptase [9], which may be necessary for infection by SARS CoV-2. A serine protease inhibitor, camostat mesylate, was recently shown to prevent entry of the virus into the lung cells of SARS-CoV-2-infected patients [6].

One prominent mechanism receiving considerable attention in SARS-CoV-2 pathogenesis is the renin–angiotensin system and its active product, angiotensin II. Angiotensin-converting enzyme 2 (ACE2) protects the lungs from acute respiratory distress syndrome (ARDS) as it breaks down angiotensin II, the pro inflammatory, vasoactive peptide that causes acute lung injury in SARS-CoV-2 patients [10]. ACE2 is also the receptor for the SARS-CoV-2 spike protein, through which the virus gains entry to host cells, and is upregulated by SARS-CoV-2-induced IFN production [11]. Patients with comorbidities are commonly treated with RAS blockers, such as angiotensin-converting enzyme inhibitors (ACEIs) or angiotensin-receptor blockers (ARBs), which is the subject of debate [12]. Mainly, in vivo studies using mice suggest ACE2 is upregulated with ACEIs/ARBs, which may possibly facilitate infection with COVID-19 [13]. Other studies suggest SARS-CoV-2 binding to ACE2 may inhibit ACE2 activity, which skews the ACE/ACE2 balance to a state of heightened angiotensin II activity leading to pulmonary vasoconstriction and inflammatory and oxidative organ damage [14]. Medical guidelines suggest continuing ACEIs/ARBs in patients with COVID-19 unless clinically indicated, and avoiding them in patients without hypertension, heart failure, or diabetes. Also, clinical trials using angiotensin II receptor antagonists are underway to assess if ACEIs/ARBs can reduce the incidence of mortality associated with COVID-19 lung injury (ClinicalTrials.gov identifiers: NCT04311177 and NCT04312009).

The role of lung MCs in COVID-19 is still unclear, as studies related to SARS-CoV-2 and human MCs suggest conflicting results, implicating them as either protective, deleterious, or innocent bystanders. Here, evidence is presented that human lung MCs are a source of renin and may be a possible reservoir for the SARS-CoV-2 virus through COVID-19-related cytokine-mediated upregulation of those receptors required for cellular infection.

## 2. Results

### 2.1. Human Lung MCs as a Source of Renin

**Figure 1:**
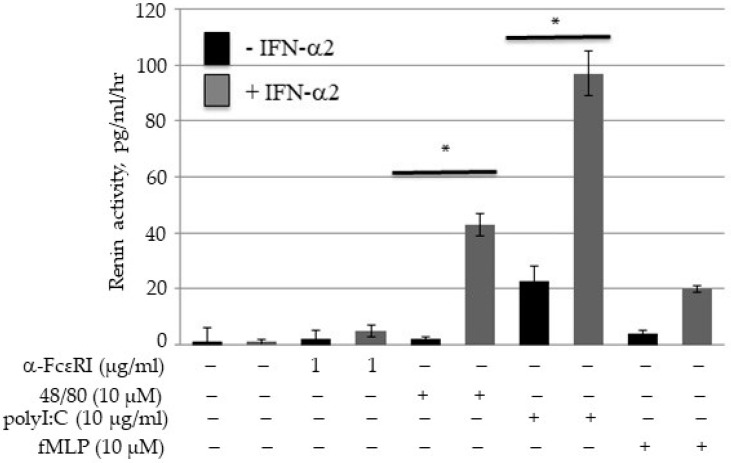
Human lung MCs as a source of renin. Human lung MCs (1 × 10^6^) were treated with (grey) or without (black) IFN (10 ng/mL) for 24 h, washed, and challenged with optimal concentrations of activator, and the amount ANG I formed (renin activity, pg/mL/h) was determined using a kit from ALPCO according to their instructions (mean ± SEM; *n* = 3; * *p* < 0.05).

Rodent MCs have been shown to be a source of renin, a precursor to angiotensin I formation [15]. We demonstrated that chymase, which is involved in angiotensin II formation [16], distinguishes two sub-types of MCs in humans [17]. It has been reported that human lung MCs and MC-like cells express renin at both the message and protein levels, and elicit the release of ANG I-forming activity [18]. However, it is not known how renin is released from the MC. To explore this mechanism, we examined renin release following interferon (generated in the lung following viral infections, including SARS-CoV-2) incubation and FcεRI and non-FcεRI stimuli. As seen in Figure 1, we detected renin activity/ANG I after double-stranded RNA (polyI:C, a synthetic mimic of viral RNA) and compound 48/80 challenges of human lung MCs. Unlike previous studies of rodent MCs [19], no renin release was observed after FcεRI-challenges of human lung MCs. Of note is the observation that a significant increase in response to these secretagogues was only observed after IFN exposure, suggesting that the viral-induced immune response directly affects lung MC functional responses.

### 2.2. Upregulation of ACE2 and TMPRSS in MCs following FcεRI and IFN Challenges

In previous work [20], we performed gene and protein microarray analysis on resting vs. FcεRI-challenged human MCs and examined genes that were up- or down-regulated. We reviewed these data after the SARS-CoV pandemic emerged to determine if human MCs upregulated those genes involved in SARS-CoV2 mechanisms of infection. As seen in Table 1, both ACE2 and TMPRSS genes are upregulated in FcεRI-stimulated human MCs. Although MCs expressed the IFN receptor (IFNAR), no upregulation was observed following an FcεRI challenge [20].

**Figure 2:**
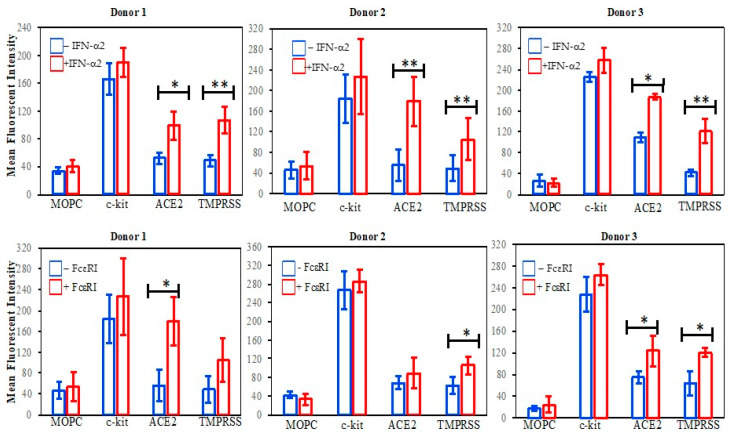
Human lung mast cells expression of SARS-CoV-2 receptors ACE2 and TMPRSS following a cytokine or FcεRI challenge. Mast cells were purified and treated with IFN-α2 (10 ng/mL) or Fab’_2_ of anti-FcεRI Ab (2 μg/mL) for 24 h. Surface expression was performed using mouse anti-human primary Abs followed by Fc-specific, FITC-conjugated anti-mouse Abs and FACs. Each condition was assessed in duplicate using non-specific mouse IgG (MOPC) as a negative control. Bars indicate SD, * *p* < 0.05 and ** *p* < 0.005.

Previous studies have demonstrated that human MCs express receptors for interferon (IFN) that mediate their function [21]. Type I IFNs are used to treat viral diseases, and clinical trials of IFN for COVID-19 patients are ongoing [22]. This cytokine is abundant in SARS-CoV-2 patients’ lungs and contributes as a first line of defense against viruses [22]. ACE2 and TMPRSS genes (which mediate the cellular entry of SARS-CoV2) are upregulated by IFN-α2 and IFN-γ [11]. We next hypothesized that ACE2 and TMPRSS genes in human lung MCs may be upregulated by FcεRI and/or IFN-α2 stimulation [11], resulting in increased surface expression. As seen in Figure 2, there was a significant (*p* < 0.05) surface upregulation of ACE2 and TMPRSS receptors after IFN-α2 incubation (Figure 2, top) in each donor examined. Significant upregulation of ACE2 was observed in two of the three donors, and in one of the three donors (ACE2, *p* = 0.072; TMPRSS, *p* = 0.063) following FcεRI stimulation (Figure 2, bottom). These are the first studies that suggest human lung MCs can upregulate the cell surface receptors that act as a reservoir for the SARS-CoV-2 virus, which has important clinical implications that may provide insight into the pathogenesis of SARS-CoV-2, differential patient susceptibility, and therapeutic strategies.

## 3. Discussion

The spike protein of SARS-CoV-2 is required for viral entry into target cells. Entry also depends on cellular proteases such as TMPRSS2. Identifying the cell subsets targeted by SARS-CoV-2 (ACE2+) and those at greatest risk of resultant infection (ACE2+TMPRSS2+) is critical for understanding the virus’ pathogenesis and the host’s defense to it. We have shown that IFN-α2 upregulates ACE2 and TMPRSS2 on the surface of human lung MCs. It has also been demonstrated that these cells produce renin in response to non-IgE-mediated stimuli.

Prior studies of rodent MCs and MC lines have suggested that they may be a source of renin, but if and how renin is released by primary human MCs is unknown. Renin is involved in the formation of ANG II through the renin–angiotensin system, and binds to the angiotensin type 1 receptor to mediate vasoconstriction. Overproduction of ANG II is involved in arrhythmias and sudden cardiac death. In patients with COVID-19, hypertension is one condition that increases morbidity and mortality, possibly due to an imbalance in ACE/ACE2 levels and/or reduced levels of ACE2 [12,13]. Initial studies demonstrated that antihypertensive drugs that target the renin–angiotensin system have no significant effect on the risk of infection and disease outcome [23]. Variations in RAAS genes have been associated with the risk of developing hypertension and cardiovascular disease, and may partly explain the heterogenous response to SARS-CoV-2 infection. Apparently contradictory, several large-scale studies found only a slightly increased incidence of COVID-19, but not of severe COVID-19, in MC-mediated asthma patients [24]. These intriguing findings suggest (counter to other studies just discussed) that MCs may have a blunting effect in SARS-CoV-2 pathogenesis, as increased MC numbers in the lung is a hallmark in asthma patients [25]. What is clear is that COVID-19 from SARS-CoV-2 infection causes a hyperinflammatory response associated with a cytokine storm by the activation of several cell types, including MCs. On the other hand, epidemiological studies suggest increased MC numbers may have a protective effect in patients with co-morbidities such as asthma. Understanding the immunopathological mechanisms of SARS-CoV-2 infection and COVID-19 disease pathogenesis may help in identifying new therapeutic options and ways to prevent infection.

**Figure 3:**
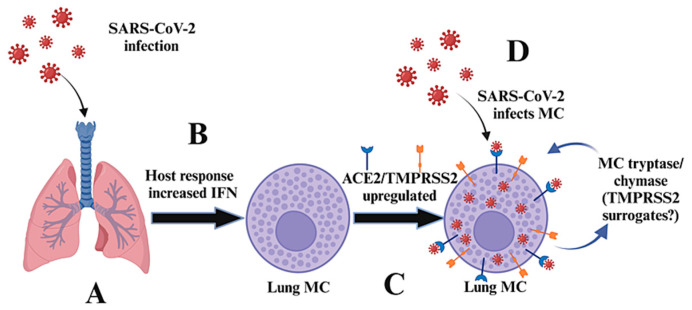
A potential unrecognized mechanism for SARS-CoV-2 replication and pathogenesis. In (**A**), SARS-CoV infects the host, inducing increases in IFN levels (**B**). The increase in lung IFN induces upregulation of ACE2 and TMPRSS2 on MCs (**C**). Lung MCs are now primed to be infected by the virus (**D**). This allows for further propagation of the virus and the alteration and/or release of potent MC mediators.

The spike protein of SARS-CoV-2 is primed by the transmembrane serine protease 2 (TMPRSS2), and antibodies against the SARS-CoV-2 spike protein offer protection against SARS-CoV-2 [11]. Little is known about whether other proteases, besides TMPRSS2, can promote the pathogenesis of SARS-CoV-2. Interestingly, the TMPRSS-related, MC-specific serine protease tryptase can mimic TMPRSS2 in aiding the viral entry of MERS (related to SARS-CoV-2) [26]. The most comprehensive study across species found ACE2 and TMPRSS2 co-expressing in cells within pneumocytes, ileal absorptive enterocytes, and nasal goblet secretory cells [11]. Further, it was shown that the ACE2 gene was upregulated by IFN-α2 (which is important for host antiviral defense) in vitro using airway epithelial cells in a species-specific way (no upregulation was observed in mouse cells) [11]. However, the authors highlighted that when analyzing single cell RNA sequence data from cells expressing low levels of transcripts such as ACE2 and TMPRSS2, underestimation of actual protein levels can be an issue, as detection inefficiencies occur; especially in this study, in which protein levels were not examined [11]. Also, the authors highlighted that pre-clinical models should be carefully analyzed, and that neither in vitro nor in vivo IFN-stimulation nor an in vivo viral challenge substantially altered ACE2 expression in mice. Indeed, as we show herein, human lung MCs have low levels of ACE2 and TMPRSS2, but are significantly upregulated at the protein level following IFN stimulation. Based on the observation that human lung MCs upregulate ACE2 and TMPRSS2 following an IFN-a2 challenge, we hypothesized that human lung MCs can be infected with strains of SARS-CoV-2, and that MC-specific protease (tryptase, chymase) may contribute to this phenomenon (Figure 3). The effect of MC chymase on propagating viral pathogenesis through protease-dependent cellular entry [27] or by converting angiotensin I to angiotensin II to regulate blood flow and blood pressure [28,29,30,31,32] may also contribute to pathogenesis.

## 4. Materials and Methods

Human lung tissue was processed under Liberty University Institutional Review Board (IRB-FY21-22 844) approval. Samples were collected under a waiver of consent for research and were de-identified to prevent any connection to patients. MCs were dispersed as described [33]. Lung MCs were cultured at 2 × 10^5^ cells/mL in 24-well plates as previously described [2]. After one week, media was replaced with AIM-V (Lonza, Switzerland) plus 80 ng/mL SCF. Half the media was replaced approximately every ten days, and cells were split when MCs were 50–75% confluent on the plate bottom. Toluidine blue staining of cytocentrifuged cells on cytospin slides was used to assess MC maturation as previously described [34].

**Renin measurement from human lung-derived MCs:** Cells (0.75 × 10^5^) were incubated with or without human IFN-α2 (10 ng/mL, R&D systems) for 48 h, washed, and incubated with IgE and non-IgE-dependent secretagogues as previously described [35,36]. The amount of ANG I formed (renin activity, pg/mL/h) was determined using a kit from ALPCO according to their instructions and the addition of angiotensinogen (mean ± SEM, *n* = 3, * *p* < 0.05). All experiments were performed in duplicate from three separate donors, and significant differences (*p* < 0.05) were performed using the Student’s *t*-test.

**Flow cytometry:** Cells (0.5 × 10^5^) were incubated with or without human IFN-α2 as above, and flow cytometry was performed using a FACS Arial III (Becton Dickenson, Franklin Lakes, NJ, USA). Briefly, mouse anti-human Abs to FcεRI, c-kit, ACE2, TMPRSS (Santa Cruz, Dallas, TX, USA) or mouse IgG isotype control MOPC (Sigma-Aldrich, St. Louis, MO, USA) were added for at least 1 h on ice, washed, and F(ab′)2-FITC-goat anti-mouse Abs (BD Biosciences, San Jose, CA, USA) was added for detection. All experiments were performed at least three times from three different donors.

## 5. Conclusions

In conclusion, these preliminary studies suggest that human lung MCs express molecules required for SARS-CoV-2 infection and secrete mediators involved in viral pathogenesis. These studies further suggest a critical role for MCs in a viral pathogenic mechanism that may have important implications for a basic understanding of the pathogenesis of the virus and potential future therapeutic strategies. The low number of samples examined in this study necessitates further research examining larger sample sizes and a direct viral challenge of MCs under conditions described above. The use of human MCs is critical, as rodent MCs do not upregulate ACE2 or TMPRSS in vitro or in vivo following cytokine stimulation, and rodent models are not prime surrogates for understanding SARS-CoV-2 pathogenesis in humans.

## Figures and Tables

**Table 1:** SARS-CoV2-related gene expression in resting vs. FcεRI-challenged human MCs.

ACE2	PATIENT	RESTING			FcεRI XL			CHANGE
		Relative gene expression		Avg	Relative gene expression		Avg	% Upregulated
	Patient 1	521	634	533	876	987	933	75%
		502	474		749	1121		
	Patient 2	453	320	415	1268	1202	1326	220%
		215	672		1302	1532		
TMPRSS	PATIENT	RESTING			FcεRI XL			CHANGE
		Relative gene expression		Avg	Relative gene expression		Avg	% Upregulated
	Patient 1	852	832	769	892	894	860	12%
		734	659		854	798		
	Patient 2	801	722	746	1001	1192	976	31%
		721	739		953	759		

## Data Availability

The original contributions presented in the study are included in the article, further inquiries can be directed to the corresponding author.
